# Adapting OptCouple to Identify Strategies with Increased Product Yields in Community Cohorts of *E. coli*

**DOI:** 10.3390/metabo15050309

**Published:** 2025-05-06

**Authors:** Nicole Pearcy, Jamie Twycross

**Affiliations:** 1 BBSRC/EPSRC Synthetic Biology Research Centre (SBRC), School of Life Sciences, The University of Nottingham, University Park, Nottingham NG7 2RD, UK; 2 School of Computer Science, University of Nottingham, Jubilee Campus, Nottingham NG8 1BB, UK

**Keywords:** OptCouple, community microbial designs, multi-directional dependent models, guaranteed product yields

## Abstract

Background: Microbesas chemical factories provide an alternative sustainable approach for producing platform chemicals. Until recently, most efforts have involved engineering heterologous pathways into a single microbial chassis to maximise its production of a target chemical. More recently, cohorts of microbes have been used to engineer microbial communities to achieve higher yields than achieved in a single chassis.

Methods: In this paper, we present a computational approach to identify sets of metabolic modifications that create stable co-dependent communities of micro-organisms and improve the chemical yield of these communities. We demonstrate our approach by designing communities which produce industrially relevant platform chemicals.

Results: We show that our approach is able to uncover metabolic engineering strategies for coupling these target chemicals to the growth of stable community cohorts.

## 1. Introduction

Microbes as chemical factories provide an alternative sustainable approach for producing platform chemicals by reducing our reliance on finite fossil fuels, whilst simultaneously reducing our carbon footprint. Until recently, most efforts have involved engineering heterologous pathways into a single microbial chassis to maximise its production of a target chemical. More recently, however, cohorts of microbes have been considered as a way of opening new opportunities for achieving higher yields [1,2]. Firstly, using multiple strains can enable the workload, that is, the metabolic burden of introducing heterologous genes, to be shared amongst the cohort and thus increase the potential product yield. Secondly, it offers opportunities to create circular economies that utilise the strengths of individual microbes in the cohort, reducing the effort for optimising, for example, substrate uptake efficiency.

Importantly, before engineering the microbial community to produce a target chemical, it is essential that the cohort is stable, such that one strain is not capable of outgrowing the other. Engineering strategies (see [3] for a comprehensive review) have been applied to create a co-dependency between strains in the cohort but rely on trial-and-error of suitable knockouts. The knockouts involved for creating a multi-directional co-dependent relationship between strains, however, are not always obvious from looking at metabolic maps of the microbes, and thus, it is advantageous to make use of genome-scale metabolic models, which allow us to search the potential design space for feasible genetic and medium modifications. In this work, we propose an adaption of the current bi-level mixed integer linear program (MILP) that is used in OptCouple [4] to identify sets of metabolic interventions that (1) create stable co-dependent communities of micro-organisms and (2) improves the chemical yield that is guaranteed by the coupling.

## 2. Materials and Methods

The following section briefly describes the mathematical optimization problem that forms OptCouple. For a more detailed description of the formulation, please refer to the original publication of Jensen et al. [4].

In [4], the authors define the growth-coupling potential as the increase in the maximum growth rate whilst allowing the target chemical to be produced. Mathematically, the growth-coupling potential is described as:

(1) $$U=\hat{f}\left(M\right)-\hat{f}\left(M*\right)$$

where *M* denotes a metabolic model with $m_{i}$ metabolites and $r_{j}$ reactions, which includes the target reaction ($r_{\mathrm{target}}$), whilst M* denotes the same metabolic model but without $r_{\mathrm{target}}$. In both *M* and M*, the biomass reaction ($r_{\mathrm{biomass}}$) is set as the objective function to maximise and is denoted by *f*. The bi-level problem can be thought of as maximising the growth-coupling potential via a set of metabolic perturbations subjected to both $M_{c}$ and $M_{c}*$, such that the growth rate of *M* and M* is optimal.

In OptCouple, the aim is to identify the optimal set of metabolic perturbations (i.e., gene knockouts, gene insertions and media supplementation) that maximises the growth-coupling potential, *U*. To achieve this, OptCouple uses an MILP-based optimisation approach, where each metabolic perturbation is represented as a binary variable, $y_{j}$, to control whether the flux is on ($y_{j}=1$) or off ($y_{j}=0$). The OptCouple mathematical formulation can be described as the following bi-level problem:

(2) $$\begin{array}{c} \begin{array}{cc} & {\mathrm{Maximise}}_{Y}:f\left(M\right)-f\left(M*\right)\\ & \mathrm{subject}\;\mathrm{to}:\\ & \;\;\;\;\;\;\;\;{\mathrm{Maximise}}_{v}:f\left(M\right)\\ & \;\;\;\;\;\;\;\;\mathrm{subject}\;\mathrm{to}:\\ & \;\;\;\;\;\;\;\;\;\;\;\;\;\;\;\;Sv=0\\ & \;\;\;\;\;\;\;\;\;\;\;\;\;\;\;\;v_{j}=0\;\forall \;j\;\in \;\left\{j|y_{j}=0\right\}\\ & \;\;\;\;\;\;\;\;\;\;\;\;\;\;\;\;v_{\mathrm{glc}-\mathrm{uptake}}\leq \;10\\ & \;\;\;\;\;\;\;\;{\mathrm{Maximise}}_{v}:f\left(M*\right)\\ & \;\;\;\;\;\;\;\;\mathrm{subject}\;\mathrm{to}:\\ & \;\;\;\;\;\;\;\;\;\;\;\;\;\;\;\;Sv=0\\ & \;\;\;\;\;\;\;\;\;\;\;\;\;\;\;\;v_{j}=0\;\forall \;j\;\in \;\left\{j|y_{j}=0\right\}\\ & \;\;\;\;\;\;\;\;\;\;\;\;\;\;\;\;v_{\mathrm{glc}-\mathrm{uptake}}\leq \;10\\ & \;\;\;\;\;\;\;\;\;\;\;\;\;\;\;\;v_{\mathrm{target}}=0\\ & y_{j}\;\in \;\left\{0,1\right\}\;\forall \;j \end{array} \end{array}$$

This bi-level problem can be thought of as maximising the growth-coupling potential, such that the growth rate of *M* and M* is also optimal. Importantly, this bi-level problem can be converted into a single linear optimization using duality theory. That is, every linear problem (also known as the primal problem) can be converted into a dual problem, such that the primal constraints become the dual variables and vice versa and the objective function is reversed, such that a maximisation problem in the primal becomes a minimisation problem in the dual. Importantly, the strong duality theorem states that if a primal problem has an optimal solution, then the optimal value in the dual problem will be equivalent. Using the duality theorem, M* can be converted to the dual problem and solved as a minimisation problem. By combining the primal problem of *M* and dual problem of M*, OptCouple can be rewritten and solved as the following single linear optimization problem:

(3) $$\begin{array}{c} \begin{array}{cc} & {\mathrm{Maximise}}_{v,\lambda ,\mu ,Y}:v_{\mathrm{biomass}}-10\cdot \mu_{\mathrm{glc}}-\mathrm{uptake}\\ & \mathrm{subject}\;\mathrm{to}:\\ & \;\;\;\;\;\;\;\;\;\;\;\;\;\;\;\;\sum_{j=1}^{\left|R\right|}s_{i,j}\cdot v_{j}=0\;\forall \;i\;\in \;N\\ & \;\;\;\;\;\;\;\;\;\;\;\;\;\;\;\;v_{j}=0\;\forall \;j\;\in \;\left\{j|y_{j}=0\right\}\\ & \;\;\;\;\;\;\;\;\;\;\;\;\;\;\;\;v_{\mathrm{glc}-\mathrm{uptake}}\leq \;10\\ & \;\;\;\;\;\;\;\;\;\;\;\;\;\;\;\;\sum_{i=1}^{\left|N\right|}\lambda_{i}^{\mathrm{stoich}}\cdot s_{i,j}+\mu_{j}=0,\;\mathrm{if}\;j\neq \mathrm{biomass}\\ & \;\;\;\;\;\;\;\;\;\;\;\;\;\;\;\;\sum_{i=1}^{\left|N\right|}\lambda_{i}^{\mathrm{stoich}}\cdot s_{i,\mathrm{biomass}}+\mu_{\mathrm{biomass}}=1,\;\mathrm{if}\;j\neq \mathrm{biomass}\\ & \;\;\;\;\;\;\;\;\;\;\;\;\;\;\;\;\mu_{j}=0\;\forall \;j\;\in \;\left\{j|y_{j}=1\right\},\;\mathrm{if}\;j\neq \mathrm{target}\\ & \;\;\;\;\;\;\;\;\;\;\;\;\;\;\;\;y_{j}\;\in \;\left\{0,1\right\}\;\forall \;j\\ & \;\;\;\;\;\;\;\;\;\;\;\;\;\;\;\;\sum_{j}^{|R_{\mathrm{native}}|}\left(1-y_{j}\right)\leq K_{\mathrm{native}}\\ & \;\;\;\;\;\;\;\;\;\;\;\;\;\;\;\;\sum_{j}^{|R_{\mathrm{native}}|}y_{j}\leq K_{\mathrm{heterologous}} \end{array} \end{array}$$

where λ represents the dual variables of the stoichiometric constraints in the primal and μ represent the dual variables of any other flux constraints in the primal (e.g., uptake constraints). To reduce the search space, constraints are added so that a maximum number of gene knockouts, insertions and supplements are allowed. A more detailed derivation of the above can be found in [4].

### 2.1. Adapting OptCouple to Community GSMs

A community genome-scale metabolic model, $M_{c}$, is constructed such that each strain is represented as a different compartment in the model, and thus have their own unique subscript on reaction and metabolite identifiers. Universal exchange reactions are added to the community model to represent the uptake of nutrients from the external environment, as well as transport reactions that allow these nutrients to be transferred to each individual strain in the cohort, for example:

$$\begin{array}{cc} EX_{{\mathrm{glucose}}_{u}} & :{\mathrm{glucose}}_{u}\to \\ r_{\mathrm{glucose}-{\mathrm{transport}}_{s_{n}}} & :{\mathrm{glucose}}_{u}\to {\mathrm{glucose}}_{s_{n}} \end{array}$$

where *u* and $s_{n}$ denote whether a metabolite is universal or specific to one of the n strains. The optimal growth rate of the community model is considered here as a linear combination of each strain’s biomass equation. For this reason, the growth-coupling potential in the community model, given by

(4) $$U=\hat{f}\left(M_{c}\right)-\hat{f}\left(M_{c}*\right)$$

corresponds to the difference between the total (weighted) growth rate of all strains in the cohort with and without cross-feeding. The objective function given in Equation (3) can therefore be written as the following for the community model:

$$\begin{array}{cc} \; & {\mathrm{Maximise}}_{v_{s_{1}},\lambda_{s_{1}},\mu_{s_{1}},Y_{s_{1}},\cdots ,v_{s_{n}},\lambda_{s_{n}},\mu_{s_{n}},Y_{s_{n}}}:\\ \; & c_{1}\cdot v_{{\mathrm{biomass}}_{s_{1}}}+\cdots +c_{n}\cdot v_{{\mathrm{biomass}}_{s_{n}}}-10\cdot \mu_{\mathrm{universal}-\mathrm{glucose}-\mathrm{uptake}} \end{array}$$

where $c_{n}$ represents the coefficients for weighting the growth rate of each strain in the objective function, $\mu_{universal-glucose-uptake}$ corresponds to the global glucose uptake of the community model and the coefficient 10 is the maximum uptake value set in the primal. Note that in order to ensure all strains are growing in the cohort, we set a minimum growth rate of 0.05 $h^{-1}$ on each strain in the community.

Cross-feeding reactions (one for each direction) are added to the model to allow the exchange of metabolites between the compartments/strains. One cross-feeding reaction is defined for every available (exchange) metabolite such that all strains produce every dummy metabolite. *n* dummy metabolites are added to the model (i.e., 1 for each strain) and included in the cross-feeding reactions as follows:

$$\begin{array}{cc} r_{m_{i},{\mathrm{crossfeeding}}_{s_{1},s_{2}}} & :m_{i,s_{1}}\to m_{i,s_{2}}+{\mathrm{dummy}}_{s_{1}}\\ & \vdots \\ r_{m_{i},{\mathrm{crossfeeding}}_{s_{n},s_{n-1}}} & :m_{i,s_{n}}\to m_{i,s_{n-1}}+{\mathrm{dummy}}_{s_{n}} \end{array}$$

Artificial transport reactions are added that consume each of the dummy metabolites. A multi-directional growth coupling (i.e., cross-feeding is essential for the growth of each strain in the community) can be achieved by adding the following constraints to the model:

$$\begin{array}{cc} r_{{\mathrm{dummy}}_{s_{1}}} & :w_{1}\cdot {\mathrm{dummy}}_{s_{1}}\to \mathrm{target}\\ & \vdots \\ r_{{\mathrm{dummy}}_{s_{n}}} & :w_{n}\cdot {\mathrm{dummy}}_{s_{n}}\to \mathrm{target}\\ EX_{\mathrm{target}} & :\mathrm{target}\to \end{array}$$

where *w* represents the stoichiometries of each dummy metabolite and can be any positive real number and provide a way of weighting the cross-feeding exchanges according to the optimal ratio between strains. Setting $EX_{\mathrm{target}}$ as the target reaction can be interpreted as finding the set of metabolic interventions that results in the largest difference in the maximum community growth rate of $M_{c}$ and $M_{c}*$, such that metabolites are allowed to exchange in both directions, at some fixed ratio, in $M_{c}$ and not allowed to in $M_{c}*$. Note, however, that a strong multi-directional relationship is only achieved for solutions where $f(M_{c}*)$ is equal to zero, since this ensures that the growth of each individual strain is zero in $M_{c}*$ and hence dependent on the cross-feeding. The mathematical formulation for the community model is as follows:

(5) $$\begin{array}{c} \begin{array}{cc} & {\mathrm{Maximise}}_{v_{s_{1}},\lambda_{s_{1}},\mu_{s_{1}},Y_{s_{1}},\cdots ,v_{s_{n}},\lambda_{s_{n}},\mu_{s_{n}},Y_{s_{n}}}:\\ & c_{1}\cdot v_{{\mathrm{biomass}}_{s_{1}}}+\cdots +c_{n}\cdot v_{{\mathrm{biomass}}_{s_{n}}}-10\cdot \mu_{\mathrm{universal}-\mathrm{glucose}-\mathrm{uptake}}\\ & \mathrm{subject}\;\mathrm{to}:\\ & \;\;\;\;\;\;\;\;\;\;\;\;\;\;\;\;\sum_{i=1}^{\left|R\right|}s_{i,j}\cdot v_{j}=0\;\forall \;i\;\in \;N\\ & \;\;\;\;\;\;\;\;\;\;\;\;\;\;\;\;v_{j}=0\;\forall \;j\;\in \;\left\{j|y_{j}=0\right\}\\ & \;\;\;\;\;\;\;\;\;\;\;\;\;\;\;\;v_{\mathrm{universal}-\mathrm{glucose}-\mathrm{uptake}}\leq \;10\\ & \;\;\;\;\;\;\;\;\;\;\;\;\;\;\;\;\sum_{i=1}^{\left|N\right|}\lambda_{i}^{\mathrm{stoich}}\cdot s_{i,j}+\mu_{j}=0,\;\mathrm{if}\;j\neq \mathrm{biomass}\\ & \;\;\;\;\;\;\;\;\;\;\;\;\;\;\;\;\sum_{i=1}^{\left|N\right|}\lambda_{i}^{\mathrm{stoich}}\cdot s_{i,\mathrm{biomass}}+\mu_{\mathrm{biomass}}=1,\;\mathrm{if}\;j\neq \mathrm{biomass}\\ & \;\;\;\;\;\;\;\;\;\;\;\;\;\;\;\;v_{j}=0\;\forall \;j\;\in \;\left\{j|y_{j}=0\right\}\\ & \;\;\;\;\;\;\;\;\;\;\;\;\;\;\;\;\mu_{j}=0\;\forall \;j\;\in \;\left\{j|y_{j}=1\right\},\;\mathrm{if}\;j\neq \mathrm{target}\\ & \;\;\;\;\;\;\;\;\;\;\;\;\;\;\;\;y_{j}\;\in \;\left\{0,1\right\}\;\forall \;j\\ & \;\;\;\;\;\;\;\;\;\;\;\;\;\;\;\;\sum_{j}^{|R_{\mathrm{native}}|}\left(1-y_{j}\right)\leq K_{\mathrm{native}}\\ & \;\;\;\;\;\;\;\;\;\;\;\;\;\;\;\;\sum_{j}^{|R_{\mathrm{native}}|}y_{j}\leq K_{\mathrm{heterologous}}\\ & \;\;\;\;\;\;\;\;\;\;\;\;\;\;\;\;\sum_{j}^{|R_{\mathrm{native}}|}y_{j}\leq K_{\mathrm{crossfeeding}}s_{1}\\ & \;\;\;\;\;\;\;\;\;\;\;\;\;\;\;\;\vdots \\ & \;\;\;\;\;\;\;\;\;\;\;\;\;\;\;\;\sum_{j}^{|R_{\mathrm{native}}|}y_{j}\leq K_{\mathrm{crossfeeding}}s_{n} \end{array} \end{array}$$

Notably, the number of cross-feeding reactions between strains can be controlled using the binary vector *Y*. Candidate solutions identified for creating uni- or multi-directional relationships within cohorts can be included as optional constraints in a second step for producing some target chemical. Alternatively, however, the cross-feeding exchanges can be left unconstrained and selected by the algorithm if they provide an advantage to the cohort for coupling the target chemical to the community growth rate, as discussed in the following section.

### 2.2. Adapting OptCouple to Maximise the Minimum Possible Product Yield in Community Strains

The above extension of OptCouple to a community setting provides a tool for identifying metabolic engineering strategies that couple a target chemical to growth within the community. This approach, however, currently does not consider the yield of the target when selecting solutions, and therefore often results in strategies that have extremely low production rates of the target. In this work, we therefore modified OptCouple to find solutions that have a growth-coupling potential with guaranteed yield above some threshold, *X*, which is set by the user. The higher the threshold is set, the more likely it is that cross-feeding reactions will be identified that are beneficial to target production (as demonstrated by our case studies in the next section). The mathematical formulation given in Equation (2) therefore becomes the following for the community model with chemical production greater than *X*:

(6) $$\begin{array}{c} \begin{array}{cc} & {\mathrm{Maximise}}_{Y}:f\left(M_{c}\right)-f\left(M_{c}*\right)\\ & \mathrm{subject}\;\mathrm{to}:\\ & \;\;\;\;\;\;\;\;{\mathrm{Maximise}}_{v}:f\left(M_{c}\right)\\ & \;\;\;\;\;\;\;\;\mathrm{subject}\;\mathrm{to}:\\ & \;\;\;\;\;\;\;\;\;\;\;\;\;\;\;\;Sv=0\\ & \;\;\;\;\;\;\;\;\;\;\;\;\;\;\;\;v_{j}=0\;\forall \;j\;\in \;\left\{j|y_{j}=0\right\}\\ & \;\;\;\;\;\;\;\;\;\;\;\;\;\;\;\;v_{\mathrm{glc}-\mathrm{uptake}}\leq \;10\\ & \;\;\;\;\;\;\;\;\;\;\;\;\;\;\;\;v_{\mathrm{target}}\leq \;1000\\ & \;\;\;\;\;\;\;\;{\mathrm{Maximise}}_{v}:f\left(M*\right)\\ & \;\;\;\;\;\;\;\;\mathrm{subject}\;\mathrm{to}:\\ & \;\;\;\;\;\;\;\;\;\;\;\;\;\;\;\;Sv=0\\ & \;\;\;\;\;\;\;\;\;\;\;\;\;\;\;\;v_{j}=0\;\forall \;j\;\in \;\left\{j|y_{j}=0\right\}\\ & \;\;\;\;\;\;\;\;\;\;\;\;\;\;\;\;v_{\mathrm{glc}-\mathrm{uptake}}\leq \;10\\ & \;\;\;\;\;\;\;\;\;\;\;\;\;\;\;\;v_{\mathrm{target}}<X\\ & y_{j}\;\in \;\left\{0,1\right\}\;\forall \;j \end{array} \end{array}$$

Here, $v_{\mathrm{target}}<X$ constrains $M_{c}*$ to have a maximum flux of *X* through the target reaction. This optimisation problem can now be interpreted as finding the set of metabolic interventions that maximises the difference in growth rates of $M_{c}$ and $M_{c}*$ when flux is allowed through the target reaction above and below the target threshold, respectively. Any combination of metabolic interventions that is found ensures that the target *X* yield is guaranteed as the minimum expected yield when the micro-organism is operating at the maximal growth rate. Using strong duality theory as before, the bi-level problem can be rewritten as a linear optimization problem as follows:

(7) $$\begin{array}{c} \begin{array}{cc} & {\mathrm{Maximise}}_{v_{s_{1}},\lambda_{s_{1}},\mu_{s_{1}},Y_{s_{1}},\cdots ,v_{s_{n}},\lambda_{s_{n}},\mu_{s_{n}},Y_{s_{n}}}:\\ & c_{1}\cdot v_{{\mathrm{biomass}}_{s_{1}}}+\cdots +c_{n}\cdot v_{{\mathrm{biomass}}_{s_{n}}}-10\cdot \mu_{\mathrm{universal}-\mathrm{glucose}-\mathrm{uptake}}-X\cdot \mu_{\mathrm{target}}\\ & \mathrm{subject}\;\mathrm{to}:\\ & \;\;\;\;\;\;\;\;\;\;\;\;\;\;\;\;\sum_{i=1}^{\left|R\right|}s_{i,j}\cdot v_{j}=0\;\forall \;i\;\in \;N\\ & \;\;\;\;\;\;\;\;\;\;\;\;\;\;\;\;v_{j}=0\;\forall \;j\;\in \;\left\{j|y_{j}=0\right\}\\ & \;\;\;\;\;\;\;\;\;\;\;\;\;\;\;\;v_{\mathrm{universal}-\mathrm{glucose}-\mathrm{uptake}}\leq \;10\\ & \;\;\;\;\;\;\;\;\;\;\;\;\;\;\;\;\sum_{i=1}^{\left|N\right|}\lambda_{i}^{\mathrm{stoich}}\cdot s_{i,j}+\mu_{j}=0,\;\mathrm{if}\;j\neq \mathrm{biomass}\\ & \;\;\;\;\;\;\;\;\;\;\;\;\;\;\;\;\sum_{i=1}^{\left|N\right|}\lambda_{i}^{\mathrm{stoich}}\cdot s_{i,\mathrm{biomass}}+\mu_{\mathrm{biomass}}=1,\;\mathrm{if}\;j\neq \mathrm{biomass}\\ & \;\;\;\;\;\;\;\;\;\;\;\;\;\;\;\;v_{j}=0\;\forall \;j\;\in \;\left\{j|y_{j}=0\right\}\\ & \;\;\;\;\;\;\;\;\;\;\;\;\;\;\;\;\mu_{j}=0\;\forall \;j\;\in \;\left\{j|y_{j}=1\right\},\;\mathrm{if}\;j\neq \mathrm{target}\\ & \;\;\;\;\;\;\;\;\;\;\;\;\;\;\;\;y_{j}\;\in \;\left\{0,1\right\}\;\forall \;j\\ & \;\;\;\;\;\;\;\;\;\;\;\;\;\;\;\;\sum_{j}^{|R_{\mathrm{native}}|}\left(1-y_{j}\right)\leq K_{\mathrm{native}}\\ & \;\;\;\;\;\;\;\;\;\;\;\;\;\;\;\;\sum_{j}^{|R_{\mathrm{native}}|}y_{j}\leq K_{\mathrm{heterologous}}\\ & \;\;\;\;\;\;\;\;\;\;\;\;\;\;\;\;\sum_{j}^{|R_{\mathrm{native}}|}y_{j}\leq K_{\mathrm{crossfeeding}}s_{1}\\ & \;\;\;\;\;\;\;\;\;\;\;\;\;\;\;\;\vdots \\ & \;\;\;\;\;\;\;\;\;\;\;\;\;\;\;\;\sum_{j}^{|R_{\mathrm{native}}|}y_{j}\leq K_{\mathrm{crossfeeding}}s_{n} \end{array} \end{array}$$

In summary, in $M_{c}$, we allow the flux to be unconstrained through the target chemical, whereas in $M_{c}*$ we allow flux but not above the threshold *X*. The objective function is looking for a strategy where the growth rate in $M_{c}$ is greater than the growth rate in $M_{c}*$, where the only difference in the models is this constraint on this target reaction. The only way that this objective can be any different from zero, is therefore by finding perturbations that force flux through the target at values greater than *X*, otherwise, any value less would be achievable also in $M_{c}*$, and thus, the objective function is still zero. This is the same as OptCouple; however, OptCouple does not allow any flux through the target, and therefore, the objective function of F(M)–F(M*) can be high even when the target flux is very low. Our approach, therefore, enforces a minimal yield.

## 3. Results

### 3.1. Coupling 2,3-Butanediol to Community Growth

As a proof of principle, we tested the ability of the adapted MILP to predict stable community growth-coupling strategies for producing a minimum yield of the industrially relevant platform chemical 2,3-butanediol (2,3-BD) in a consortia of two *E. coli* strains. 2,3-BD has many applications in industry, including its use as a solvent, a precursor to polymers and resins, and in high-quality aviation fuel [5,6]. The expected growth of the product is expected to reach USD 220 million by 2027 [7], whilst its downstream products are predicted to reach a worth of USD 43 billion [7]. The identification of sustainable and renewable routes to produce 2,3-BD using yeasts and bacteria has therefore become a promising alternative to the high-cost petrol-based approaches currently being used [8]. Synthesis of 2,3-BD has been engineered in single *E. coli* strains [9], and here we use 2,3-BD synthesis to illustrate how synthesis could also be engineered in a community and therefore potentially take advantage of community properties (e.g., robustness, improved carbon utilisation).

**Figure 1:**
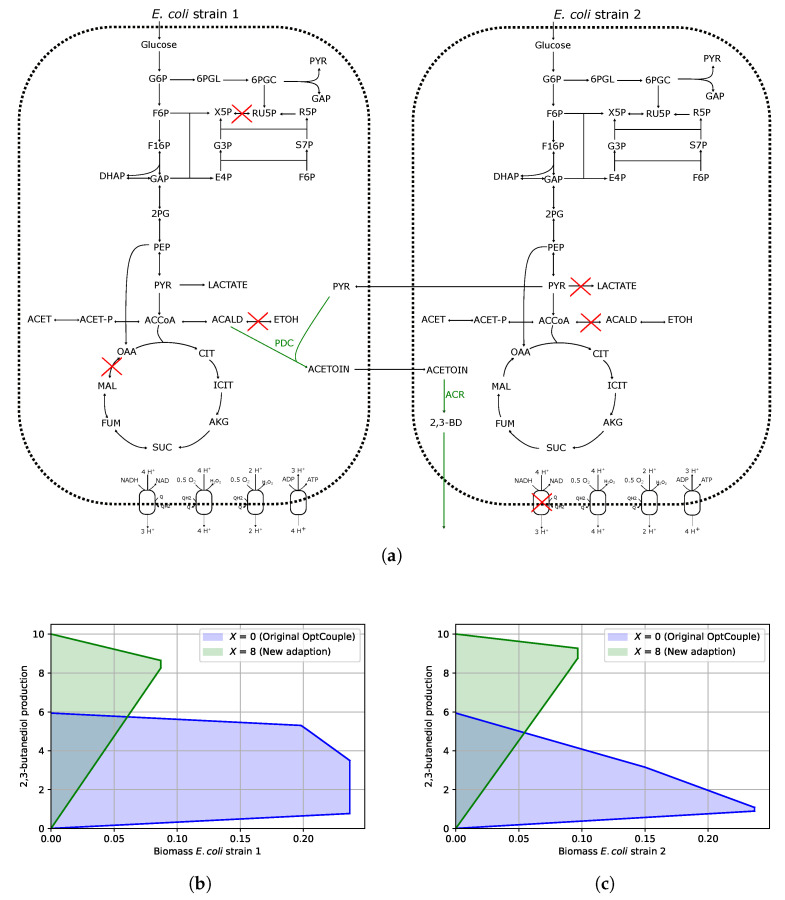
Overview of the strategy identified with the adapted OptCouple for finding growth couples of 2,3-butanediol in community cohorts in two *E. coli* strains. (**a**) Metabolic network demonstrating the predicted design for coupling 2,3-BD to community growth. (**b**,**c**) Production envelopes showing the minimum and maximum 2,3-BD production rate (mmol · ${\mathrm{gDCW}}^{-1}\cdot$ $h^{-1}$) as the biomass is increased in each strain. The green shaded regions are the results from our new adapted OptCouple, whereas the blue shaded regions are the results when running the original OptCouple on the community model. Red crosses indicate the reactions knocked out by our algorithm.

To predict a community-level 2,3-butanediol–biomass-coupled knockout strategy, we first constructed the community GSM (as described in the Materials and Methods section) using the reduced metabolic model EColiCore [10], which was downloaded from the BiGG database [11]. The metabolite 2,3-BD is not native to *E. coli*, and therefore, we added acetoin reductase (ACR in Figure 1a), which catalyses the conversion of acetoin to 2,3-butanediol, to one *E. coli* strain, and added a pyruvate decarboxylase (PDC in Figure 1a) known to convert acetaldehyde and pyruvate into acetoin to the other *E. coli* strain. The adapted version of OptCouple was then run on this model whilst allowing a maximum of two cross-feeding reactions to be active (one from strain 1 to strain 2 and one in the opposite direction) and a total of 10 knockouts native to the community model. We ran the simulation under anaerobic conditions (i.e, oxygen uptake fixed at 0 mmol · ${\mathrm{gDCW}}^{-1}\cdot$ $h^{-1}$) and selected glucose as the feedstock with a maximum uptake rate fixed at 10 mmol · ${\mathrm{gDCW}}^{-1}\cdot$ $h^{-1}$, whilst the lower bound of the growth rate in each strain was fixed at 0.05 $h^{-1}$. To determine the value of *X* (i.e., minimum product yield) to use, we calculated the maximum theoretical yield and then gradually decreased the value until we found a solution within a reasonable time frame. All algorithm implementations, simulation constraints and parameter settings are provided and documented in the Jupyter Notebooks available at the DOI given in the data availability statement for this paper.

After running the algorithm for just an hour, we were able to find a promising knockout strategy that resulted in 58% of the carbon being redirected to 2,3-butanediol in the optimal solution, which, notably, is 93% of the maximum theoretical yield in this community model. The strategy involved knocking out three reactions from one of the *E. coli* strains, an additional three reactions knocked out in the other *E. coli* strain and the bidirectional cross-feeding of pyruvate and acetoin, as detailed in Table 1 and illustrated in Figure 1a. The knockouts *ALCD2x* and *MDH* in strain 1 block the sinking of NADH via ethanol and succinate production, respectively, whilst the knockout RPE results in 6-phosphogluconate dehydrogenase (Gnd) being activated and thus improves the supply of reducing equivalents. Importantly, over-expressing Gnd has previously been shown to increase other NADH-dependent biochemicals, including succinate [12]. As a result of these knockouts, the only remaining route for sinking NADH is via the production of acetoin from acetyl-CoA (via PDC). However, the excess pyruvate required by PDC is not producible by strain 1 and thus becomes dependent on cross-feeding from strain 2. The knockouts in strain 2 also block pathways that sink NADH, including the 2-step pathway to ethanol, lactate production and the electron transport chain, and importantly, result in 2,3-BD biosynthesis becoming the optimal route for NADH production. The inability of strain 2 to produce acetoin leads to a bidirectional dependency between the strains, and thus creates a stable community, such that the strains grow at a very similar optimal rate.

Importantly, this strategy leads to 2,3-butanediol being 100% growth coupled to the community growth rate (i.e., the maximum combined biomass flux of each strain), as well as each individual growth rate (Figure 1b,c green shaded area). For comparison purposes, we also ran the community adaption of OptCouple without including the constraint that guarantees a minimal yield of the target chemical and found a considerable difference in the growth-coupling potential. As shown in Figure 1b,c, the solution space of our new approach suggests the minimum yield of 2,3-BD has improved almost 10-fold compared to the old version of OptCouple (blue shaded region). We also show that the two cross-feeding metabolites are coupled to the growth of each strain (i.e., the minimum production rate is always greater than zero and follows a similar trend to the production rate of 2,3-BD).

### 3.2. Coupling Butanol to Community Growth

To test the ability of the algorithm to predict strategies that have also been validated in vivo [13], we used the algorithm to search for knockouts that coupled butanol production to biomass in a stable community cohort. In [13], the authors developed a butanol-producing strain of *E. coli* (BuT-3E), which they obtained by constructing a synthetic pathway using an acetoacetyl-CoA transferase (*atoDA*) and an aldehyde-alcohol dehydrogenase (*adhE2*). Additionally, the authors deleted the native lactate dehydrogenase (*ldhA*), fumarate reductase (*frdA*), phosphate acetyltransferase (*pta*), pyruvate dehydrogenase (*poxB*) and ethanol producing alcohol dehydrogenase (*adhE*). Importantly, this strain is able to produce butanol when supplemented with butyrate and the concurrent production of acetate. A second butyrate-producing strain (BuT-8L-ato), which requires acetate supplementation, was therefore used to create a complete butyrate–acetate cycle between the two strains. The but-8L-ato strain converts acetyl-CoA to butyrate using the heterologous genes β-ketothiolase (*phaA*), 3-hydroxybutyryl-CoA dehydrogenase (*hbd*), crotonase (*crt*), trans-enoyl-CoA reductase (*ter*) and acetoacetyl-CoA transferase (*atoDA*), and is also deficient of the native genes *ldhA*, *frdA*, *pta*, *poxB* and *adhE*. This combination of gene insertions, deletions and cross-feeding between the two strains leads to butanol being coupled to the growth of both strains.

**Figure 2:**
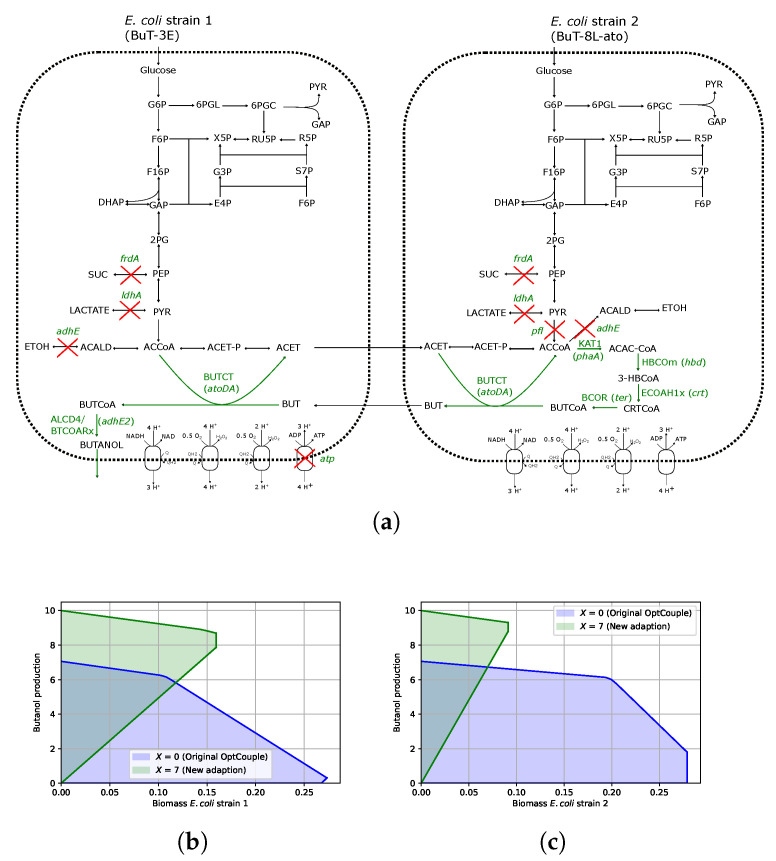
Overview of the strategy identified with the adapted OptCouple for finding growth couples of butanol in community cohorts in two *E. coli* strains. (**a**) Metabolic network demonstrating the predicted design for coupling butanol to community growth. (**b**,**c**) Production envelopes showing the minimum and maximum butanol production rate (mmol · ${\mathrm{gDCW}}^{-1}\cdot$ $h^{-1}$) as the biomass is increased in each strain. The green shaded regions are the results from our new adapted OptCouple, whereas the blue shaded regions are the results when running the original OptCouple on the community model. Red crosses indicate the reactions knocked out by our algorithm.

To attempt to reproduce this strategy, we first constructed the two strains BuT-3E and BuT-8L-ato by adding the heterologous pathways to the reduced metabolic model, EColiCore, as illustrated in Figure 2. A community model was then constructed, as described in the Materials and Methods section, that allowed cross-feeding between the two strains. Note that we allowed for any metabolite that is able to transported outside the system in EColiCore to be involved in cross-feeding. The adapted OptCouple algorithm was then run for 1 h whilst allowing for 10 knockouts and one cross-feeding reaction in each direction (i.e., cross-feeding from strain 1 to strain 2 and vice versa). We again ran the simulation under anaerobic growth conditions with glucose as the feedstock with a maximum uptake rate fixed at 10 mmol · ${\mathrm{gDCW}}^{-1}\cdot$ $h^{-1}$, whilst the lower bound of the growth rate in each strain was fixed at 0.05 $h^{-1}$. As before with 2,3-BD, we found the maximum theoretical yield of butanol in this community metabolic model and then gradually decreased this value until we found a feasible solution within a reasonable time limit.

After running the algorithm for just an hour, we were able to find a promising knockout strategy that resulted in 52% of the carbon being redirected to butanol in the optimal solution, which is 84% of the maximum theoretical yield in this community model. Importantly, this in silico design suggested six out of the eight knockouts listed in [13] and predicted the same cross-feeding metabolites (acetate and butyrate) between the two strains, as shown in Table 1 and illustrated in Figure 2. The consistent knockouts between the in silico and in vivo designs were again blocking NADH production for redirecting flux towards the NADH-sinking butanol pathway. The only minor differences between the designs was that the model suggested was also blocking the ATP synthase reaction in strain 1 (BuT-3E), a competing sink of NADH, as well as the pyruvate formate lyase (*pfl*) in strain 2 (BuT-8L-ato). Additionally, according to the model, the phosphotransacetylase (pta) reaction does not carry any flux, given the other knockouts are implemented, and thus does not require knocking out. Despite these minor differences, the high similarities between the model predictions and the design in [13] demonstrate that our adapted version of OptCouple can be applied to suggest promising metabolic engineering strategies for coupling a target chemical to the growth of a stable community cohort.

Furthermore, we again compared the strategy produced via our new adapted OptCouple (Equation (7)), which guarantees a minimum yield (*X*) of the target chemical, against the standard OptCouple algorithm adapted to a community model Equation (6). Importantly, as shown in Figure 2b,c, the solution space of our new approach shows butanol production is 100% coupled to each strain’s biomass production, whereas the strategy produced using the original approach is only coupled at growth rates above 0.22 $h^{-1}$ for the biomass in strain 1 and not growth coupled at all for the biomass in strain 2. This result highlights the potential our new adaption to the MILP of OptCouple in Equation (7) has for finding strong and stable growth couples in community cohorts.

## 4. Discussion

In this paper, we show that the adapted version of OptCouple we have developed can be applied to suggest promising metabolic engineering strategies for coupling a target chemical to the growth of a stable community cohort. The growth rate of the community strains is a linear combination of each individual biomass—ideally, a non-linear function would be used that removes any strategy that results in one strain out-competing the other. Currently, we set a constraint where the growth rate of each individual strain is at least 0.05 $h^{-1}$ to avoid one strain not growing at all but with the fluxes still being utilised to produce metabolites that are then cross-fed into the optimal strain. Introducing the threshold in Equation (7), however, does appear to improve the stability of the strains, since it now tries to redirect high amounts of carbon into the target and therefore tries to identify strategies where neither one of the strains is capable of wasting carbon as other products, as was shown in our two example cases.

Currently, we only show examples where knockouts are identified and insert the heterologous reactions into the model prior to running the algorithm. The number of variables in a community model, however, becomes extremely large, and thus, the algorithm struggles to identify reaction insertions. In future work, it would therefore be useful to develop approaches to try to reduce the search space to make this feasible in a reasonable time-frame. These approaches would also be useful to reduce runtime when running our approach with larger models.

One limitation of our approach is the use of a linear objective function of the growth rates of each strain in the cohort, which can lead to flux being redirected from other strains towards another strain if it leads to a higher overall growth rate. To prevent this from occurring, and ensuring all strategies discovered result in valid growth in all strains in the cohort, we fix a lower bound on the growth rate in each strain. Notably, predefining a lower bound may limit the possible strategies identified in our algorithm. To overcome these issues, a multi-level optimisation problem, such that the target chemical was optimized subject to the independent growth rate of each strain (i.e., each strain contributes an additional level in the optimization problem), could be applied. Multi-level optimization problems, however, require significantly greater computational runtime compared to bi-level problems, due to the complexity in the nested structure of the optimization, and are therefore left as a suggested future area of development.

## Figures and Tables

**Table 1:** Overview of the community growth-coupling strategies predicted by the adapted OptCouple for two target chemicals. Here, we show the predicted reaction knockouts, the metabolites involved in cross-feeding between the two *E. coli* strains, the production rate (mmol · ${\mathrm{gDCW}}^{-1}\cdot$ $h^{-1}$) of the biomass-coupled target product and the carbon yield. Note that the reaction knockouts are denoted by their BiGG identifiers and include a subscript to define which strain they are from.

Product	Knockouts	Cross-Feeding	Production Rate	Yield
2,3-butanediol	$NADH16_{ec2}$ $LDH\_D_{ec2}$ $ACALD_{ec2}$ $ALCD2x_{ec1}$ $RPE_{ec1}$ $MDH_{ec1}$	$pyruvate_{ec2}$ $acetoin_{ec1}$	8.58	57.2%
Butanol	$ALCD2x_{ec1}$ $ATPS4r_{ec1}$ $LDH\_D_{ec1}$ $MDH_{ec1}$ $PFL_{ec2}$ $ACALD_{ec2}$ $LDH\_D_{ec2}$ $MDH_{ec2}$	$acetate_{ec2}$ $butyrate_{ec1}$	7.8	52%

## Data Availability

All algorithm implementations, data and code created during this research are openly available from the University of Nottingham data repository at http://doi.org/10.17639/nott.7517 (accessed on 5th May 2025).

## Abbreviations

The following abbreviations are used in this manuscript:

MILP	Mixed integer linear program
GSM	Genome-scale model
